# Crushed and Injected Buprenorphine Tablets: Characteristics of Princeps and Generic Solutions

**DOI:** 10.1371/journal.pone.0113991

**Published:** 2014-12-04

**Authors:** Régis Bouquié, Laura Wainstein, Paul Pilet, Jean-Marie Mussini, Guillaume Deslandes, Johann Clouet, Eric Dailly, Pascale Jolliet, Caroline Victorri-Vigneau

**Affiliations:** 1 Service de Pharmacologie Clinique, Centre Hospitalier Universitaire de Nantes, Nantes, France; 2 EA 4275 Biostatistique, Pharmacoépidémiologie et Mesures Subjectives en Santé, Université de Nantes, Nantes, France; 3 INSERM (Institut National de la Santé et de la Recherche Médicale), UMR (Unité Mixte de Recherche) 791, Laboratoire d’Ingénierie Ostéo-Articulaire et Dentaire (LIOAD), Université de Nantes, Nantes, France; 4 Centre Hospitalier Universitaire de Nantes, Pôle Hospitalo-Universitaire 4, Nantes, France; 5 Laboratoire d'Anatomie Pathologique, Centre Hospitalier Universitaire de Nantes, Nantes, France; 6 Institut National de la Santé et de la Recherche Médicale (INSERM) Unité Mixte de Recherche en Santé (UMRS) 791, Laboratoire d’Ingénierie Ostéo-Articulaire et Dentaire (LIOAD), Université de Nantes, Nantes, France; 7 Centre Hospitalier Universitaire Nantes, Pôle Hospitalo-Universitaire (PHU) 7, Pharmacie Centrale, Nantes, France; 8 Clinical Pharmacology, Service de Pharmacologie Clinique, Centre Hospitalier Universitaire de Nantes, Nantes, France; 9 EA 3826 Thérapeutiques Cliniques et Expérimentales des Infections, Université de Nantes, Nantes, France; 10 Pharmacology, Service de Pharmacologie Clinique, Centre Hospitalier Universitaire de Nantes, Nantes, France; Harvard Medical School, United States of America

## Abstract

Self-injection of high-dose buprenorphine is responsible for well-described complications. In 2011, we have been alerted by unusual but serious cutaneous complication among injection buprenorphine users. A prospective data collection identified 30 cases of necrotic cutaneous lesions after injection of filtered buprenorphine solution, among which 25 cases occurred following injection of buprenorphine generics. The main goal of our study was to put forward particularities that could explain the cutaneous complications, by qualitatively and quantitatively confronting particles present in Subutex and generics solutions. We used the same protocol that injected-buprenorphine users: generic or subutex tablets were crushed in sterile water and filtered through 2 filters commonly used (cotton-pad and sterifilt). Solutions were analyzed by laser granulometry, flow cytometry and scanning electron microscopy. We have highlighted the wide variation of the quantity and the size of the particles present in solution between the two drugs after cotton-pad filtration. The proportion of particles <10 µm is systematically higher in the generic solutions than with Subutex. All of the insoluble particles found in generic solutions contain silica, whereas non- organic element was to be identified in the insoluble particles of Subutex. One skin biopsy obtained from one patient who developed a necrotic lesion after intravenous injection of filtrated solution of buprenorphine generic, shows non-organic elements. Identification of particles *in situ* enables us to confirm the presence of silica in the biopsy. Actually the monitoring of patient receiving generic of buprenorphine must be strengthened.

## Introduction

Buprenorphine is a synthetic opioid, more specifically a partial µ receptors agonist and a κ receptors antagonist. Since 1996, high dosage sublingual forms (Subutex 0.4, 2 and 8 mg) have been commercialized as maintenance treatment for major opiate dependence. Since 2006, high dosage buprenorphine is registered in the generic drugs index. In 2013, 5 laboratories were commercializing buprenorphine copies with new dosages of 1, 4 and 6 mg in addition to the already available 0.4, 2 and 8 mg dosages, since 2008. In France, buprenorphine treatment can be established by general practitioner, as opposed to methadone treatment; therefore, about 2/3 of patients treated for opioid dependence are prescribed buprenorphine, versus 1/3 of patients who are prescribed methadone [1]. Opioid substitution treatments decrease severe withdrawal syndrome, but injection of the substitution (methadone, buprenorphine) is not recommended for drug abuser. Self-injection substitution is not good for abuse treatment, and over-dose always occurred following self-injection. Indeed, it seems that almost two thirds of patients show a sustained global improvement for at least two years [2]. Shortly after it was first commercialized, we started noticing that sublingual forms of Subutex were diverted through sniffing, inhaling, injecting, administration of high doses and primo-dependence cases. In 2012, according to the OFDT (French Monitoring Centre for Drugs and Drug Addiction), the diverted share of prescribed Subutex would involve 6 to 40% of that drug, and 15% of patients would regularly self-inject buprenorphine tablets [3]. Taking into account the number of patients who were subsitutely treated by buprenorphine, the results are satisfying. However, the possibility of injecting Subutex can cause severe infectious complications: abscesses, infectious diseases (viral hepatitis, HIV, endocarditis, osteoarticular infections…) [4], along with adverse effects at the injection site: venous thrombosis, edema, necrosing ulcers, skin infections, etc.

Injection drug users have a Steribox at their disposal, which is a prevention kit that contains a sterile injection set along with a sterile dish and a sterile cotton filter. In some hosting structures, Sterifilt are also available for drug users. The purpose of these filters is not to sterilise the solution, but to eliminate insoluble particles and gaz bubbles that potentially are present in the solutions. The risks related to the presence of particles in an injectable solution are well known: from the end of the 60′s, studies on animals have shown how quantity, size, form, and chemical nature of the particles influence the damage caused by the injected solution [5], [6]. At the same period of time, the first clinical trials assessing the use of a terminal filter placed right before a perfusion injection site all reported a decrease in phlebitis [7]–[9]. Over the year 2011, a new type of complications linked to buprenorphine self-injection appeared, which alerted the center of evaluation and information on pharmacodependence (CEIP-A). These lesions were fully described by Wainstein and colleagues to be serious necrotic cutaneous lesions (red/purplish, with irregular edges, inflammatory, of approximately several centimetres), which appeared at or near the injection site [10]. 89% of patients did the injection in the upper limbs, with sometime multiple sites of injection. For 66.66% of patients the intravenous injection was used, for 16.66% the injection was intra-arterial, for 16.66% the injection was failed.

Although it is always favourable, the healing of these cutaneous lesions is only completed after a few weeks. The most severe cases required several surgical interventions. Wainstein and colleagues have identified 30 cases of cutaneous lesions since. The patients had been intravenous drug users for many years and they had never suffered from particular problems due to injection. Surprisingly, in 25 out of 30 cases, it was generic of buprenorphine that was injected [10]. At the end of 2011, the National Agency for Drug and Health Products Safety (ANSM) issued an information note for the health professionals. At the beginning of 2013, the ANSM issued an information key point on the health consequences of Subutex and generics injection misuse.

**Figure 1:**
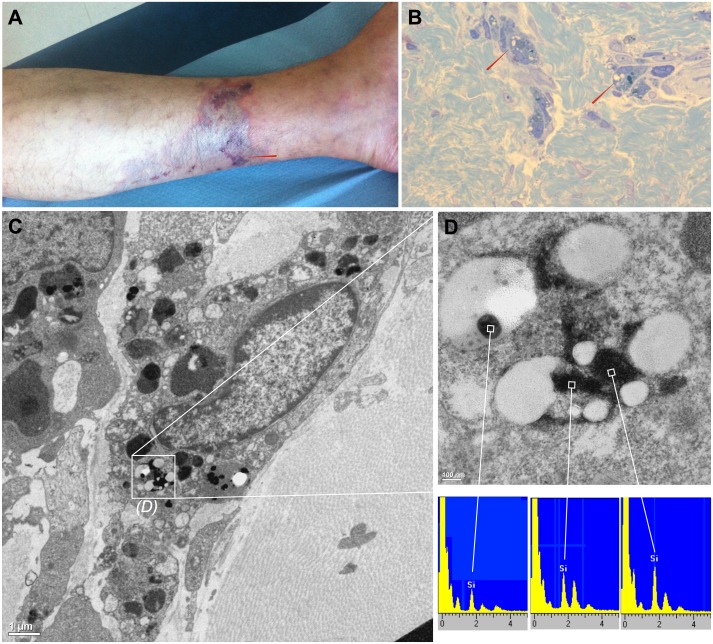
Macroscopic view, microscopic view and particles analysis of a representative cutaneous lesion obtained from the same patient. A: necrotic livedo-like dermatitis lesion from the lower left leg. The red arrow represents the injection site. B: macrophages easily distinguished by CD68 immunolabelling among a perivascular inflammatory infiltrate exhibited few refringent material (red arrows). C: transmission electron microscopy, we have observed non organic very dense particles, without epoxy permeation but with either a round shape compatible with silica or with a laminated aspect compatible with silicate. D: High magnification of figure 1C inlay by transmission electron microscopy and energy-dispersive x-ray spectroscopy spectra analysis. Particles primarily identified as very dense and non organic contain silica (Si).

Among all 30 cases described, only one skin biopsy was obtained for a patient, which was a self-injector of generics of buprenorphine without any other drugs. The figure 1A show a macroscopic picture a representative cutaneous lesion, and the injection site (red arrow). The dermatologic diagnosis was livedo-like dermatitis with necrotic lesion. The biopsy of this lesion showed refringent material in the perivascular inflammatory infiltrate compatible with non-organic particles (figure 1B). Transmission electronic microscopy showed very dense particles compatible with inorganic insoluble particles (figure 1C). Spectral analyses of these particles by energy-dispersive x-ray spectroscopy show the presence of silica (figure 1D: magnification of figure 1C inlay). Silica has been observed, in most of the particles suspected.

The increasing incidence of cutaneous complications due to injected buprenorphine generics prompted us to study *in vitro* solutions that were self-injected by the patients. The main goal of this study was to put forward particularities that could explain the cutaneous lesions, by qualitatively and quantitatively confronting Subutex and generics solutions.

## Materials and Methods

### Chemicals and reagents

At the time of this study, all sublingual generic formulation available in France (Arrow, Biogaran, Mylan, Sandoz and Teva) was produced from the same factory and contained the same qualitative raw material. Sterile injection material used was that contained in the Steribox II Kit (Apothicom, Paris, France): a sterile single-use aluminium container for mixing (Stericup), a cotton pad or a 10 µm pore size filter (Sterifilt) for filtering, syringes (1 ml) and sterile water. Solutions were prepared using Subutex 8 mg (RB Pharmaceuticals, Massy, France) or buprenorphine 8 mg generic as follows: one sublingual tablet was dissolved in 1 ml sterile water at room temperature in the Stericup, mashed with the syringe’s piston and filtered through one of the 2 filters. Six different solutions were obtained. To make reading easier, they will be identified as follows: NFS for *Non-Filtered Subutex*; CFS for *Cotton-Filtered Subutex*; SFS for *Sterifilt-Filtered Subutex*; NFG for *Non-Filtered Generic*; CFG for *Cotton-Filtered Generic* and SFG for *Sterifilt-Filtered Generic*. When 2 tablets were necessary, 2 identical solutions were independently prepared and extemporaneously mixed. Talc and magnesium stearate used as an indicator for the elementary analysis were purchased by the Cooper laboratory (Melun, France).

### Ultra Performance Liquid Chromatography

The liquid chromatography system consisted of a Waters Acquity UPLC instrument with a photodiode array detector (Waters, Milford, USA). Chromatographic separation was performed on an Acquity UPLC BEH C18 1.7 µm, 2.1×150 mm column with the corresponding guard column (Waters). Buprenorphine calibration curve (0, 5, 7.5, 10, 15 and 20 ng/mL) was constructed from buprenorphine standard solution purchased from LGC Standard (Toronto Research Chemical, North York, Canada). The solution obtained by tablets dissolution was analysed after dilution with water (1/10 000^e^).

### Flow cytometry (FACS)

The different insoluble particles subsets were assessed by using flow cytometry (LSR II, BD Biosciences, San Jose, CA). FSC (Forware SCatter) and SSC (Side Scatter) parameters can look at a mix of cells or events and distinguish them from one another based off of size. The FSC parameter (relative size) is a measurement of the amount of the laser beam that passes around the cell. This gives us a relative size for the cells using a known control. We have diverted this technology to determine the relative size of the suspended particles in the various studied solutions. BD Fluorosphere (4.2, 10 and 15 µm) were used as known standard size. Data were stored and analyzed using the flow cytometer’s operating software (BD FACSDiva, V6.1.3, BD Biosciences).

### Laser granulometry

Size distributions of particles in solution were determined using a laser diffraction granulometer (LS230 Analyser, Beckman Coulter). The size measurement is based on the relationship between the light intensity and particle diameter. The size was calculated in terms of the distribution of volume percentages on the basis of the Fraunhofer approximation with an overall sizing range going from 0.4 µm to 2000 µm. Analysis of the six solutions were performed by the Lab-Service SA company (La Roche Vineuse, France) on diluted aqueous solutions (final volume 125 mL). Each sample was measured 10 fold.

### Scanning electron microscopy (SEM)

For electron microscopy and elementary analysis the 6 different solutions were treated as follows: 2 identic solutions of each condition (NFS, CFS, SFS, NFG, CFG and SFG) were independently prepared and mixed before being diluted in 2 L of ultrapure water. The solutions were filtered with Durapore polyvinylidene difluoride, 0.22 µm, 47 mm, membrane (Millipore, Molsheim, France). Membranes were carefully dissected in their center and then gold/palladium- or carbon-coated on a Desk III (Denton Vacuum, Moorestown, USA). Size, form and composition of particles disposed on the membrane were analyzed. SEM studies were performed with backscattered electrons (Leo 1450 VP, Zeiss, Oberkochen, Germany). The compositional particles analysis was determined by energy dispersive system X-ray analysis (Inca Oxford instrument, UK).

### Ethic statement

Following the French Health Authorities decision to implement a specific and prospective national case collection of these cutaneous complications, Nantes clinical pharmacology department was named to coordinate the investigations. Because French law does not impose a reviewing by an ethics committee for a survey of health professionals, no further institutional review board was consulted neither for this article nor for Wainstein’s publication [10]. Nevertheless, the patient depicted in figure 1, was informed that data can be used for scientific use, and has signed a written informed consent form for publication, and all patients described in Waintein’s article were informed that data they have provided can be used for scientific used and publication, unless they expressly oppose to it. Briefly, health professionals practicing in drugs users care center have been aware of these cutaneous problems and encouraged to report cases of unusual complication in bubrenorphine user.

### Statistical analysis

Statistical analyses used Graphpad Prism 6.0 software (Graphpad Software, La Jolla, USA). The non-parametric Mann-Whitney test was used to identify differences in granulometry study.

## Results

### Pharmaceutic formulation

The galenic analysis, which compares the 2 formulations, underlines differences in the presence of insoluble excipients, under the dissolution conditions that we used. Subutex consists of only one magnesium stearate insoluble excipient, whereas 3 insoluble excipients are present in the generic form: magnesium stearate, talcum and colloidal anhydrous silica. In water, corn starch creates a viscous suspension, which cannot be considered as particular. Quantitatively speaking, a generic tablet weighs 110 mg, versus 400 mg for a Subutex tablet.

### Buprenorphine quantification

**Figure 2:**
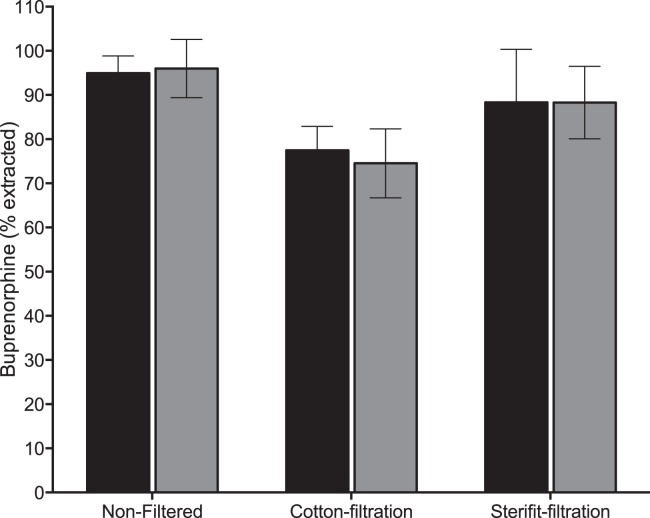
Extractibility of buprenorphine from sublingual tablets. One tablet of buprenorphine 8 mg (Subutex or generic) was diluted in 1 ml of sterile water. Drug concentration was monitored with chromatographic method before filtration or after cotton-filtration or Sterifilt-filtration for the Subutex or its generic. The volume of liquid obtained after filtration was assessed by weight difference between the vacuous syringe and full syringe. The concentration was measured after dilution of 1/10 000 in sterile water. The concentration value was transformed in percentage of the nominal dosage (8 mg).

Preparing the solutions according to the protocol used by drug users rapidly appeared to be the critical stage of our study. Filtrating a solution requires some skill, and it appeared to be a source of important interindividual variability. In order to characterize this variability, we based our study on a quantifiable parameter by systematically dosing the buprenorphine in the solutions produced for the study. Figure 2 represents the absolute quantity of buprenorphine, collected in each of the 6 conditions mentioned in Material and Methods. For a considered condition, there are no significant statistical differences between the two drugs. The observed differences depend more on filtration conditions than on tested drug. Cotton pad use significantly decreases the quantity of collected buprenorphine, whichever drug is considered. This results can be explained by the important void-volume of cotton pads –measured concentrations are similar to other conditions, but the collected volume is smaller.

### Granulometry studies

#### Laser granulometry

**Figure 3:**
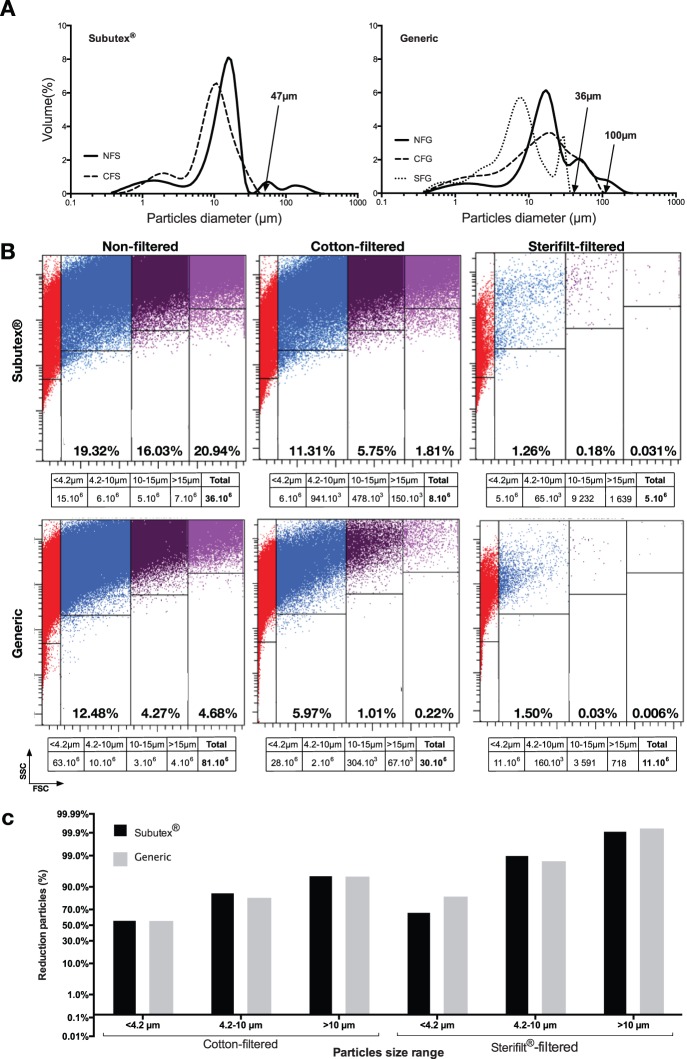
Granulometry studies. A: Laser granulometry analysis. Laser light scattering particle size histogram of particles in solution. Values are reported for volume-weighted analyses. B: Flow cytometry particles analysis. Size range and number of particles present in solution were evaluated by flow cytometry. Calibrated beads were used to define 4 gates <4.2 µm, 4.2–10 µm, 10–15 µm and >15 µm. Percentage of particles was indicated in the corresponding gate. Absolute number of particles is indicated in the tables under the corresponding dotplot. C: Percentage reduction in the number of particles after filtration measured by flow cytometry (taking the average total number of particles in unfiltered injections to be 100%). For each size range, the percentage of reduction between unfiltered and cotton-filtered or sterifilt-filtered solution was calculated. Values are reported in probability axis.

The laser granulometry technology is dedicated to particle size distribution analysis. The bar charts are represented on Figure 3A for Subutex and generics. The NFS and NFG solutions showed a multimodal and polydispersed distribution of particle sizes with respectively a mean diameter of 21.29 µm and 27.62 µm and a median diameter centered on 14.45 µm and 17.81 µm (Mann–Whitney test, *p* = 0.14). The presence of particles which size is superior to 100 µm for the 2 buprenorphine formulations should be noted. The granulometric profiles of the 2 drug solutions are therefore significantly identical. However it should be noted that the share of particles >30 µm is higher with the generic, and that size populations are more homogeneous with Subutex. Filtering generic solutions enables the elimination of particles which diameter is superior to respectively 100 µm and 36 µm, with the cotton filter and the Sterifilt. For CFB and CFS solution, the mean diameter was respectively of 20.36 µm and 11.30 µm (Mann–Whitney test, *p* = 0.0497). With Subutex, no particle which size is superior to 47 µm can be identified after cotton filtration. The granulometric analysis on the SFS solution could not be performed for lack of a large enough number of particles.

#### Flow cytometry

The analysis of the various solutions by flow cytometry confirms the presence of an important quantity of suspended particles (figure 3B). When not filtered, a Subutex solution mainly contains particles which size is over 4.2 µm (56.29%), whereas a generic solution mainly contains particles which size is smaller than 4.2 µm (78.57%).

Overall, the absolute number of particles measured is systematically higher in the generic solution than in Subutex (figure 3B). After filtration the absolute number of particles with a diameter <10 µm and in particular <4.2 µm remains higher in the generic solution than in Subutex, while Subutex solution contains systematically higher number of >10 µm diameter particles than generic. Figure 3C represents how the number of particles shrinks under the various conditions. For Subutex, cotton filters enable to reduce by 85.28% the proportion of particles which size ranges from 4.2–10 µm, and by 95.26% the proportion of particles which size is >10 µm. For the buprenorphine generic, this particle number reduction by cotton filtration was respectively by 81.48% and 94.87%. The same comparison made between non- filtered solutions and Sterifilt use shows a reduction by 99.05% and 98.41% of the 4.2–10 µm particles proportion, and by 99.92% and 99.94% of the >10 µm particles proportion, respectively for Subutex and generic.

#### Scanning electron microscopy

**Figure 4:**
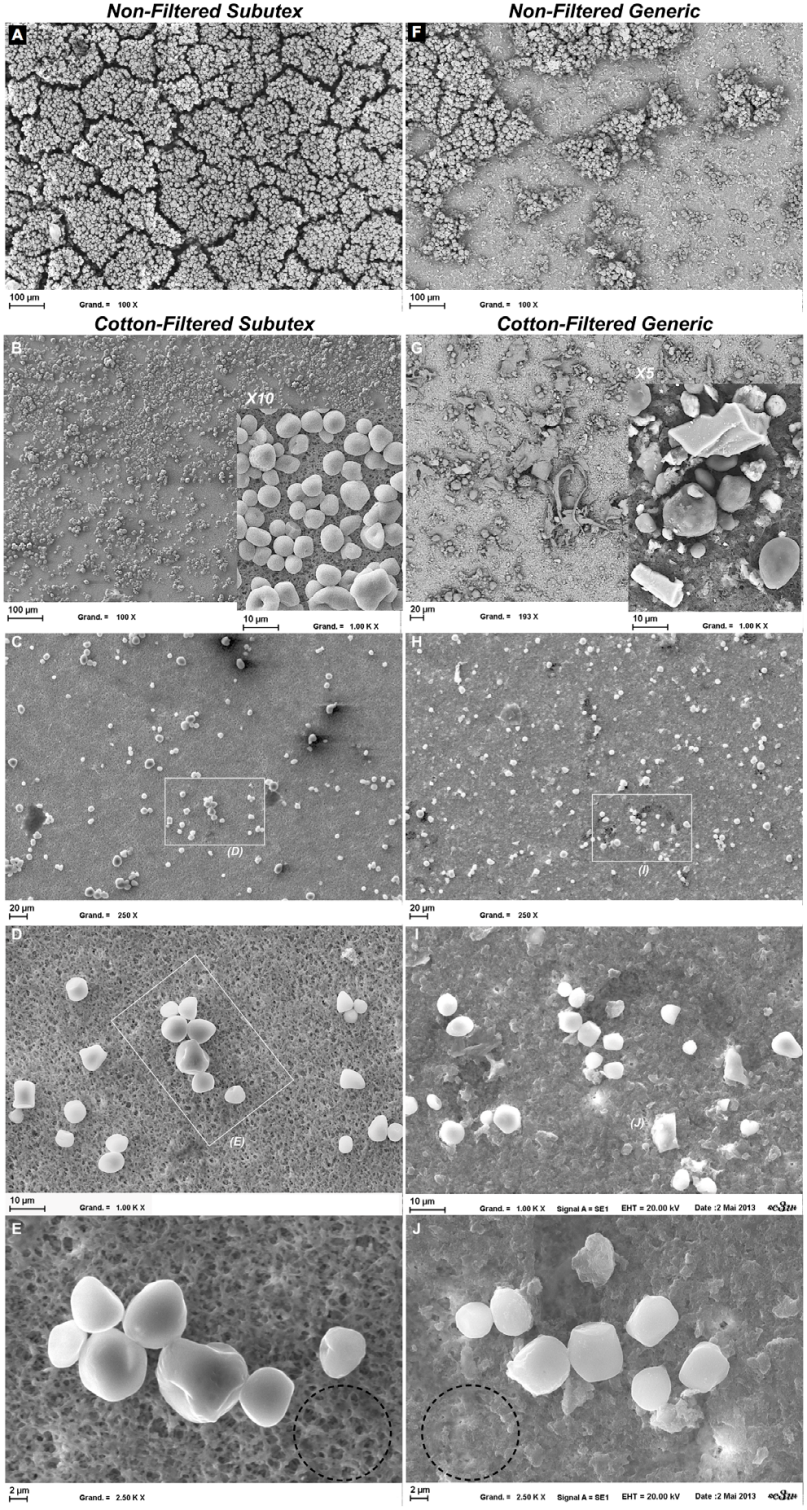
Scanning electron microscope images of insoluble particles of Subutex (left panel) and generic (right panel). A and F: solution before filtration; B–E and G–J: solution after cotton-filtration. B and G image (and inlay) were analysed after gold/paladium-coating. C–E and H–J: the magnification of the scanning microscope being varied from 250 to 2500 after carbon-coating.

SEM has been used to visualise the size and shape of particles present in solution. The main part of corn starch contained in the 2 types of tablets was eliminated diluting each solution in ultrapure water. The goal was to remove the interferences related to corn starch crystallization. Under these conditions, only the insoluble particles from the various solutions got on the filtration membrane. Images of insoluble particles present in solution before filtration show a higher particular density in NFS than in NFG (figures 4A and 4F). The SEM images of NFS show that the dominant materials were almost uniform in size and shape. The SEM images of NFG show at least 2 different types of material: the first population was almost uniform in size and shape, whereas the second one was much smaller and much more heterogeneous in terms of shapes, more like fragments. After cotton filtration, and whatever the drug, particle density strongly decreases compared to the non filtered condition. In the case of Subutex, the particles found are identical to those of NFS, in terms of size and shape. For the generic, several populations that were not retained by the cotton filter could be identified. It includes a population with homogeneous sizes and shapes, with rounded and very refringent particles, which size is ≤10 µm (figures 4H, I and J). It also includes the population of very small particles, which cover both the first particle population (figure 4G) and the free surface of the membrane, therefore virtually hiding all the pores (figures 4J dashed line circle). Alongside these 2 populations, certain areas of the membrane enable the identification of some rare particles which size is >10 µm, and of cubic or flat shape (figure 4G + zoom). Although it is rarely found in the CFG condition, this population is never found in the CFS condition. After a Sterifilt is used, the particles are almost absent whichever drug is considered. The very rare particles that are present on the filtration membrane do not show differences between SFS and SFG. The identified fragments in the NFG and CFG condition do not show.

#### Particles analysis

**Figure 5:**
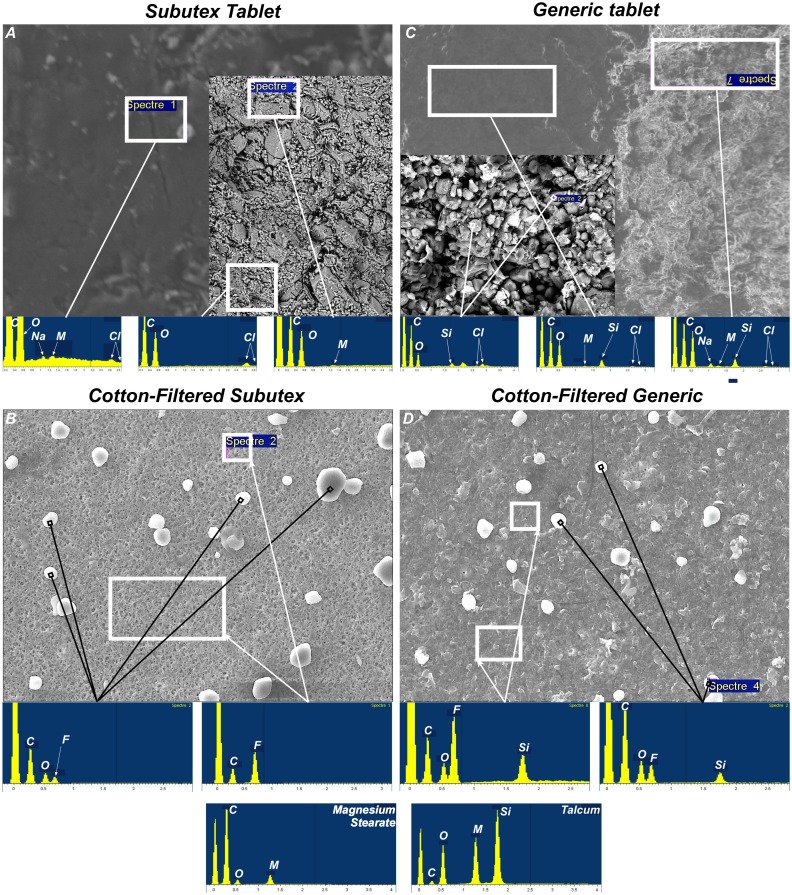
Energy-dispersive x-ray spectroscopy spectra of Subutex (left panel) and generic (right panel). White frame indicates the particles and areas analyzed. For all spectra, the large peak to the left of the carbon (C) peak is background noise, and the peak of fluor (F) is from the membrane filter. Typical spectrum of particles found inside the tablets of Subutex (A and inlay) and generic (C and inlay) showing only C, O (oxygen) and small Na (sodium), Mg (magnesium) and Cl (chloride) peaks for the 2 drugs plus silica (Si) only for the generic. Typical spectrum of insoluble particles found in CFS (B) and CFG (D). The 2 lower spectrums show peaks obtained with pure pharmaceutical magnesium stearate and talcum.

Figure 5 shows scanning electron microscopy images and energy-dispersive x-ray spectroscopy spectra for Subutex (tablets and CFS) and generic (tablets and CFG). The analysis of the CFG condition puts forward the presence of silica, both in big insoluble particles (indicated by a black square) and in the background of the filter covered with “nanoscopic” particles and “fragments” (area circumscribed by a white square). For the CFS condition, no element other than C, O and F is detected. The systematic presence of the fluorine peak originates in the very nature of the filtration membrane that is used (polyvinylidene difluoride). Analyzing the edge of the tablets (tablets sliced in 2) reveals a strong abundance of C and O whereas Mg is only detected with difficulty, whether with Subutex or generic. The absence of magnesium detection in the insoluble particles in the CFG and CFS conditions is probably due to the small amount of magnesium present in the 2 types of tablets, combined with insufficient sensibility of the technique. The main difference this analysis reveals is the presence of silica in the majority of CFG insoluble particles, and the absence of it in the CFS condition. The origin of silica (talc or colloidal anhydrous silica) cannot be precisely determined. However, these results confirm the presence of insoluble particles of different nature in the 2 types of solution.

## Discussion

Buprenorphine is one of the non-injectable drugs most diverted by drug users. Per os, buprenorphine undergoes an intense first-pass hepatic metabolism that is responsible for a bioavailability of approximately 20%. The absolute sublingual bioavailability of buprenorphine can reach 30% to 55%, depending on subjects [11]. A self-injecting drug significantly increases the administered doses, and therefore increases felt effects but also the involved risks. In France, the risk reduction policy associated to drug consumption by injectable route showed its value in the reduction of overdose death prevalence and of infectious diseases (HIV, CHV…). The main actions that were taken over the last 30 years were based on reducing the infectious risk, through authorizing over-the-counter syringe sales in pharmacies, and then through providing prevention kits (Steribox). These devices have evolved depending on the epidemics that the drug users were facing: VIH, HBV and HCV. Beyond the infectious risk, other worrying problems are associated to insoluble particles injection: phlebitis, pulmonary embolisms, « puffy hand » syndrom … [12]–[17]. In order to prevent these complications from occurring, filtrating the injected solutions has become necessary. After the end of the 90s, different types of filters have been provided for users – sterile cotton filters available in the Steribox and the Sterifilt which were provided in the Reception and Harm Reduction Support Centres for drug users (CAARUD), and in other low demand threshold structures or harm reduction structures. The cotton filters are relatively easy to use, but they present 2 major drawbacks: they let through big particles in the solution (possibly cotton fibres), and present an important void volume, which causes a substance loss leading to an increased filter misuse (selling, sharing, reusing, « squeezing »). As the Sterifilt presents a 10 µm cut-off, they retain the majority of big particles and have a very low dead volume, but are more difficult to use. Determining buprenorphine « extractability » had up to now never been studied in real use conditions. The quantity of buprenorphine retained by the cotton filters is superior to that of Sterifilt, translating *in fine* into an inferior injected doses (after cotton-pad filtration), compared to Sterifilt. These results can be compared to those obtained with heroin [18]. However, buprenorphine is an excellent candidate to injection since whatever the filtration conditions may be, at least 70% of the dosis is retrieved and can therefore be injected. The main question we have wanted to answer is the origin of the cutaneous necrotic lesions, mainly livedo-like dermatitis (LLD), observed almost exclusively during the injection of the generic [10]. Presently, LLD pathogenesis has not completely been solved. The first cases of Nicolau syndrom occurred after intramuscular injection of bismuth salt for the treatment of syphilis dates back to the twentieth century [19]. Most of cases of LLD have been reported after intramuscular injection of non steroidal anti-inflammatory, antibiotic (penicillin, aminoglycosides) or glucocorticoids drugs, and more recently by self injection of etanercept [20]–[25]. Therefore, these necrotic skin lesions always appear after an injection (arterial, peri-arterial or peri-nervous), and are variably associated with a necrosis and/or embolism/ischemia [26]–[34]. Three reports of LLDs after buprenorphine injection have been previously reported and confirmed by histological findings [35]–[37]. All cases took place in the context of intra-arterial injection. Skin biopsies showed extravascular or intravascular foreign bodies associated with inflammatory infiltrates. The features of these foreign bodies were typical of starch particles. Recently, Hornez *et* al. reported a rare case of a necrosis of the penis glans occurred after buprenorphine subcutaneous injection, and showed that ischemia was like a chemical burn with various levels of lesions and was also related to starch [14]. Potier et al. identified two main mechanisms involved in the origin of necrotic LLD: embolization of starch particles and ulcerations related to chronic dermohypodermic inflammation [35]. Schneider et al. also reported that livedoid and necrotic skin lesions were likely due to the thrombosis caused by the excipients and that local endothelial inflammation contributed to the lesions [36]. These results are consistent with the skin biopsy of the patient depicted in figure 1: dermatologists identified necrotic lesion, and biopsy reveals thrombosis, perivascular inflammation, non-organic refringent particles and particles containing silica. The main hypothesis is that a vascular mechanism could be involved. In this physiopathological context, we have oriented our research towards the detection of particles potentially present in the solutions that drug users can self-inject. Because standard anatomy and physiology textbooks report that the minimum capillary lumen measures between 4 and 8 µm in diameter [38], [39], the 4.2 µm limit has been chosen so to highlight only the particles that are capable of blocking human micro capillaries. Moreover, this cut-off is in accordance with the European Pharmacopeia concerning injectable preparations. Detecting these particles in the various tested solutions is not easy. Indeed, although the different pharmacopeia (European, American…) precisely describes which controls should be performed on active substances and on certain excipients, these recommended techniques cannot be used in the context of our study. In aqueous solutions, granulometric studies require important dilution; for this work, the solutions had to be diluted in a final volume of 125 mL. This obligation causes reduced sensibility, which explains the absence of results for the SFS condition (figure 3A). That is why we have diverted the flow cytometry from its usual application, in order to study the number of particles and their size range. Combining these 2 approaches allowed us to apprehend the distribution of insoluble particles. The total number of particles is systematically higher with the generic (figure 3B) with a majority of particle size <10 µm for the generic solution and >10 µm for the Subutex solutions whatever the size and the filtration type. These results are in accordance with the work of Roux et al. which assessed the efficiency of the Sterifilt [40]. The second step of this work was to identify the nature of the particles in solution. Although the infrared spectroscopy and the scanning electronic microscopy are reference techniques, the nature of solutions makes it impossible to exploit the results. The main obstacle is due to the large amount of corn starch present is both drugs. In aqueous solution, this excipient transforms into a opaque and viscous colloidal solution, called starch dressing, which crystallizes after it has dried and covers the other particles, which makes it hard or even impossible to interpret the analyses. In order to eliminate corn starch, the solutions were diluted before being filtered on a 0.22 µm membrane, thus enabling the retention of insoluble particles only. Under these analytical conditions, the difference in appearance of the insoluble particles retained on the filtration membrane is obvious. The generic particles size and heterogeneous shape contrast with the Subutex particle’s homogeneity. This characteristic is present both before and after cotton filtration (figure 4). This difference is even stronger when we take a look at the filtrating membrane pore visibility: they are almost completely blocked by the insoluble particles present in the CFG solutions, whereas they are apparent with the CFS solutions (figure 4E versus 4J, dashed line circle). These results confirm the data obtained by flow cytometry and by laser granulometry: the CFG condition presents a larger proportion of particles which size is inferior to 4.2 µm than the CFS condition. The heterogeneity of these particles is also apparent on SEM images of the section surface of an untampered tablet, that is to say, before any « misuse » has occurred. The SEM data obtained after Sterifilt filtration are not displayed because they are not exploitable given how extremely rare the apparent particles are on the filtration membrane surface. Therefore, after having diluted a buprenorphine tablet, only the Sterifilt seems to be capable of retaining the whole of insoluble particles, even when their size is inferior to the filter sieve. The presence of particles with extreme sizes is not found in SEM, whereas laser granulometry and flow cytometry both highlight particles which size is superior to 10 µm. This discrepancy is probably linked to the detection systems, which use light diffraction (laser granulometry and flow cytometry). These techniques cannot establish a distinction between a small particles aggregate and a sole particle. Indeed, the presence of large particles under the cotton filtration conditions is probably due to this limitation. However, should we consider this aggregation as artefactual or as « physiological » ? Several arguments seem to strengthen the reality of these aggregates.

In the first place, the insoluble excipients used in the generic are particles that bear many apolar groups on their surface. In aqueous solution, when 2 particles’ surfaces meet, the water separing them is ejected, making it easier for particles to aggregate. This physico-chemical property enables us to explain why particles for which size is inferior to 10 µm, are retained by the Sterifilt, since the main part of the filtration membrane pores are free (as confirmed by SFG solutions). After cotton filtration, the aggregates are not retained and pass through: they can be seen on the filtration membrane (Figure 4). The dashed line circles on the figure 4 show the filtration membrane pores blocked for CFG (figure 4J) whereas they are free for the CFS condition (figure 4E). The second argument that confirms the reality of these aggregates is about the experimental design used for flow cytometry. The samples analyzed by this technique are the closest to reality: the CF sample analysis only required a very small additional dilution. Therefore, it is very likely that the biggest particles highlighted by flow cytometry correspond in fact to aggregated particles. The last step of this work is about analyzing the nature of insoluble particles that were not retained in the cotton filter. We were expecting to highlight the following chemical elements: magnesium for Subutex, silica and magnesium for the generic. The spectral analysis did not bring out the presence of magnesium is the insoluble particles visualized in the CFG and CFS conditions. This result is probably related to the small quantity present in both types of tablets, combined with insufficient sensibility of the technique which does not enable us to put forward elements which abundance is <0.5%. However, the presence of silica seems to be ubiquitous for the CFG condition: the whole of analysed particles contain silica, including the fragments that cause the filtrating membrane to be blocked. The source of this silica could be the colloidal anhydrous silica, such as talc present in the generic. Under used analytical conditions, the origin of particles containing silica is impossible to determine.

However, these results remain surprising, leading to questions concerning the nature of apparent insoluble excipients. Contrary to active substances, there is not a specific pharmaceutical excipient industry: most of the time, it is only a transfer from an alimentary or cosmetic use, for example, to a pharmaceutical use. Patricia Rafidison, who represents the International Pharmaceutical Excipients Council and who was the National Pharmacy Academy’s guest at the time of a thematic session on pharmaceutical raw materials, confessed that it is was difficult to know where the excipients came from, since sometimes the suppliers themselves did not know what pharmaceutical use could be made of their product [41].

To conclude, we have shown that the injection procedure used by injectable drug users enables the extraction of about 90% of the buprenorphine contained in the sublingual tablets available on the market. The differences in galenic formulations between Subutex and its generics are also present in the solutions that users could self-inject. After using a cotton pad, there are many more insoluble particles, and they present an average size that is inferior in generic buprenorphine solution than in Subutex solution. After cotton filtration, we can also observe an important population of particles which size is inferior to 1 µm in the generic buprenorphine solutions, but not in Subutex solutions (figure 4E and 4J). All of the insoluble particles found in generic buprenorphine solutions after cotton filtration contain silica, whereas no mineral element was to be identified in the insoluble particles of Subutex.

Because of the skin biopsy was originally intended for pathology diagnostic, precise chemical identification of particles contained silica remain impossible. Nevertheless the particles identified in CFG solutions (figure 5D) and the very dense particles contained silica identified in the skin biopsy (figure 1D) might be the same. A precise chemical and structural identification of particles *in situ* should enable us to confirm this link.
